# Lithium-Ion-Conducting Ceramics-Coated Separator for Stable Operation of Lithium Metal-Based Rechargeable Batteries

**DOI:** 10.3390/ma15010322

**Published:** 2022-01-03

**Authors:** Ryo Shomura, Ryota Tamate, Shoichi Matsuda

**Affiliations:** 1 Department of Creative Engineering, National Institute of Technology, Tsuruoka College, 104 Sawada, Inooka, Tsuruoka 997-8511, Japan; SHOMURA.Ryo@nims.go.jp; 2 Center for Green Research on Energy and Environmental Materials, National Institute for Material Science, 1-1 Namiki, Tsukuba 305-0044, Japan; 3 NIMS-SoftBank Advanced Technologies Development Center, National Institute for Material Science, 1-1 Namiki, Tsukuba 305-0044, Japan

**Keywords:** lithium metal, separator, lithium-ion-conducting ceramics, lithium battery, lithium-oxygen battery

## Abstract

Lithium metal anode is regarded as the ultimate negative electrode material due to its high theoretical capacity and low electrochemical potential. However, the significantly high reactivity of Li metal limits the practical application of Li metal batteries. To improve the stability of the interface between Li metal and an electrolyte, a facile and scalable blade coating method was used to cover the commercial polyethylene membrane separator with an inorganic/organic composite solid electrolyte layer containing lithium-ion-conducting ceramic fillers. The coated separator suppressed the interfacial resistance between the Li metal and the electrolyte and consequently prolonged the cycling stability of deposition/dissolution processes in Li/Li symmetric cells. Furthermore, the effect of the coating layer on the discharge/charge cycling performance of lithium-oxygen batteries was investigated.

## 1. Introduction

With the growing demands for electric vehicles and renewable energy, there is a strong need to further increase the energy density of current lithium-ion batteries. Lithium metal is often called an ultimate anode material due to its high theoretical capacity (3860 mAh g^−1^) and low electrochemical potential (−3.04 V vs. the standard hydrogen electrode) [1,2,3]. However, the highly reactive lithium metal also leads to continuous electrolyte decomposition, which results in the formation of a thick solid electrolyte interface (SEI) and the accumulation of dead lithium during charging and discharging processes. These processes cause significant degradation of cycling performance. In addition, the formation and growth of lithium dendrites could induce short-circuit and thermal runaway of cells, posing a serious safety concern for the commercialization of lithium metal batteries.

For the practical application of lithium metal anode, there have been many recent attempts to suppress the formation and growth of dead lithium and lithium dendrites. Examples include the development of new liquid electrolytes [4,5,6,7,8], electrolyte additives [9,10], functional separators [11,12,13,14,15], organic and inorganic solid-state electrolytes [16,17,18,19,20,21], artificial SEI layers [22,23,24,25,26,27], and 3D anode structures [28,29,30,31]. Among them, the modification of the commercial separator is a promising and scalable strategy for realizing lithium metal batteries with high energy density, such as Li-sulfur and Li-oxygen batteries. Ceramic particles including Al_2_O_3_, SiO_2_, and TiO_2_ have often been coated onto polyethylene (PE) and polypropylene membrane separators to improve the mechanical strength, thermal stability, and wettability [32,33,34]. However, a coat of insulating ceramic fillers generally increases the interfacial resistance between the electrolyte and the electrode, which sometimes degrades the cell performance.

One promising way to overcome this problem is to replace the insulating ceramic fillers with Li-ion-conducting solid electrolytes [35,36,37]. In this study, a facile and scalable blade coating method is used to apply an inorganic/organic composite solid electrolyte layer to the commercial PE membrane separator in order to enhance the stability at the electrolyte–Li metal interface. This solid electrolyte layer is composed of a doped lithium aluminum titanium phosphate (LATP) glass ceramic powder (LICGC from Ohara Inc., Kanagawa, Japan), polyethylene oxide (PEO), and a Li salt (LiTFSI). The PEO/LiTFSI layer acts as the binder of the LICGC particles, as well as the lithium conducting polymer electrolyte layer. The electrochemical performance of the LICGC/PEO/LiTFSI-coated PE separator is evaluated using a Li/Li symmetric cell configuration. The coated separator shows an improved interfacial resistance between the electrolyte and the Li metal, and consequently, it prolongs stable cycling during the Li deposition/dissolution processes. Furthermore, the effect of the composite coating layer on the charge/discharge performance of lithium-oxygen batteries is investigated.

## 2. Materials and Methods

### 2.1. Materials

The powdered lithium-ion-conducting glass-ceramics (LICGC ^TM^, average particle diameter 400 nm) were obtained from Ohara Inc. (Kanagawa, Japan) and used as received. PEO (average Mv ~200,000) and lithium bromide (LiBr) were purchased from Sigma-Aldrich (St. Louis, MO, USA). Tetraethylene glycol dimethyl ether (TEGDME, water content < 10 ppm), lithium bis (trifluoromethanesulfonyl) imide (LiTFSI), and lithium nitrate (LiNO_3_) were purchased from Kishida Chemical Co., Ltd. (Osaka, Japan) LiNO_3_ and LiBr were dried at 120 °C under vacuum before use. Acetonitrile was purchased from Wako Pure Chemicals (Osaka, Japan). In all experiments, the electrolyte was a solution of 0.5 M LiTFSI, 0.5 M LiNO_3_, and 0.2 M LiBr in TEGDME.

### 2.2. Preparation and Characterization of LICGC/PEO/LiTFSI-Coated Separator

First, PEO (2.0 g) and LiTFSI (1.0 g) were dissolved in acetonitrile (7.0 g). Then, 1.0 g of LICGC was added to 1.0 g of the solution and mixed using a conditioning mixer (THINKY AR-100, THINKY, Tokyo, Japan). The viscosity of the slurry was adjusted by further adding acetonitrile. The solid content of the resulting slurry was ca. 60 wt%. This slurry was blade-coated on one side of the PE separator (W-SCOPE Corporation, Tokyo, Japan), dried overnight at room temperature, and then further dried at 50 °C under vacuum overnight. The mass loading of the coating layer is approximately 0.5 mg/cm^2^. The morphology of the separators was characterized by scanning electron microscopy (SEM; JSM-7800F, JEOL, Tokyo, Japan) and energy dispersive spectroscopy (EDS; Oxford detector, Oxford Instruments, Abingdon, Oxon, UK). The cross-sectional SEM sample was prepared using the focused ion beam technique (FIB; SMF-200, Hitachi, Tokyo, Japan). The contact angle measurements were performed with a Drop Master DM 300 (Kyowa Interface Science Co., Ltd. Saitama, Japan). The chemical change of the samples was analyzed by XPS (Axis Ultra, Kratos Analytical Co., Trafford Park, Manchester, UK) with monochromated Al Kα X-rays (hν = 1486.6 eV).

### 2.3. Assembly and Electrochemical Measurements of Li/Li Symmetric Cell

The cells were assembled in an argon-filled glovebox (UNICO, Ibaraki, Japan). The Li/Li symmetric cells consisted of two Li metal discs (diameter: 16 mm, thickness: 0.4 mm; Honjo Metal Co., Ltd., Osaka, Japan) separated by two PE separators (diameter: 19.5 mm). The amount of electrolyte in each cell was 40 µL. When using the LICGC/PEO/LiTFSI-coated PE separator, the coated side was in contact with the Li metal. Prior to the cycling tests, electrochemical impedance spectroscopy analysis was carried out using a VMP3 potentio/galvanostat (Bio-Logic Science Instruments, Grenoble, France) at a perturbation amplitude of 15 mV over a frequency range of 10^6^–10^0^ Hz at room temperature. Galvanostatic cycling tests of the Li/Li symmetric cells were carried out at 30 °C with cut-off voltages of −1.0/1.0 V vs. Li/Li^+^ using a charge/discharge system (HJ1010SM8C; Hokuto Denko Co. Ltd., Tokyo, Japan). Initially, the cells were conditioned at a current density of 0.1 mA/cm^2^ for 1 h with three cycles, 0.1 mA/cm^2^ for 10 h with one cycle, and 0.2 mA/cm^2^ for 10 h with one cycle. Subsequently, the charge/discharge tests were performed at a current density of 0.4 mA/cm^2^. The Li surface after cycling was observed by SEM (VE-9800, Keyence, Osaka, Japan).

### 2.4. Preparation of Porous Carbon Positive Electrode

A homogeneous slurry was prepared using 75 wt% of Ketjen black (KB; Lion Specialty Chemicals Co., Ltd., Tokyo, Japan, EC600J), 5 wt% of single-walled carbon nanotubes (OCSiAl, TUBALL, Luxembourg, average diameter: 1.6 nm, average length: 5 µm), 5 wt% of carbon fiber (Nippon Polymer Sangyo Co., Ltd., Osaka, Japan, CF-N, average fiber diameter: 6 µm, average length: 3 mm), 15 wt% of PAN, and NMP as a solvent. This slurry was blade-coated, and the prepared sheet sample was immersed in methanol. After drying at 80 °C for 10 h, the sample was treated at 230 °C for 3 h in the atmosphere and 1050 °C for 3 h in N_2_ with a rate of 10 °C/min and a gas flow rate of 800 mL/min.

### 2.5. Assembly and Discharge/Charge Performance Test of Lithium-Oxygen Cell

The lithium-oxygen cells were fabricated in a dry room (water content < 10 ppm) by stacking the lithium metal foil (20 mm × 20 mm × 0.1 mm, Honjo Metal Co., Ltd., Osaka, Japan), the separator (22 mm × 22 mm × 0.02 mm), the KB-based carbon electrode (20 mm × 20 mm), and the gas-diffusion layer consisting of an array of carbon fibers (overall thickness 110  µm, fiber diameter ~10  µm, TGP-H-030, Toray, Tokyo, Japan). For the electrolyte injection into carbon electrodes, the vacuum impregnation method was adopted. In addition, the electrolyte of 2.5 µL/cm^2^ was dropped into the separator. The confining pressure of the cell was controlled at approximately 100 kPa. Repeated discharge/charge test was performed (TOSCAT, Toyo System Co., Ltd., Fukushima, Japan) with a capacity limitation of 4.0 mAh/cm^2^ and cutoff voltage of 2.0 V/4.5 V. The current density during discharge and charge was set to 0.4 and 0.2 mA/cm^2^, respectively.

## 3. Results and Discussion

### 3.1. Morphology of Separator Surface

**Figure 1:**
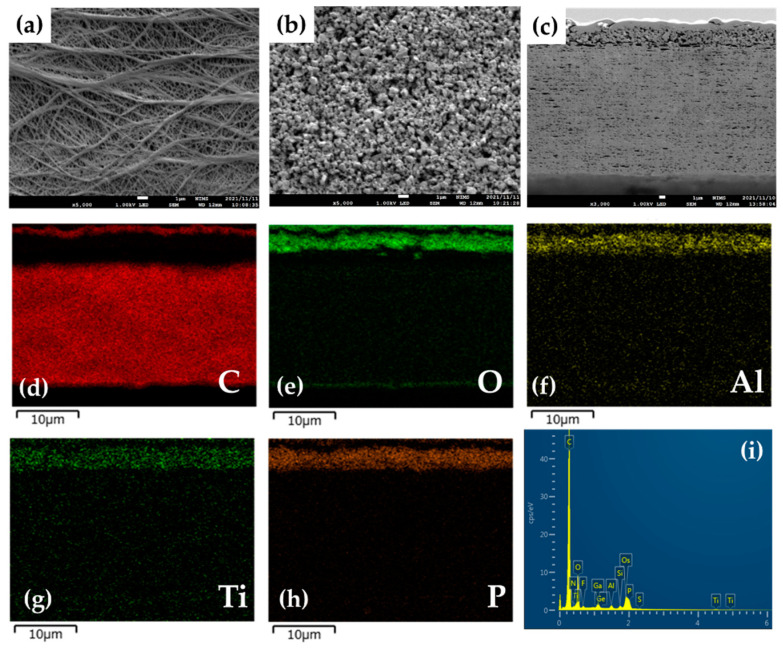
SEM and EDS images of the PE separator with and without coating. Surface SEM images of (**a**) the as-provided PE separator and (**b**) the LICGC/PEO/LiTFSI-coated PE separator. (**c**) Cross-sectional SEM image of the LICGC/PEO/LiTFSI-PE separator. (**d**–**i**) EDS mapping of the LICGC/PEO/LiTFSI-PE separator.

**Figure 2:**
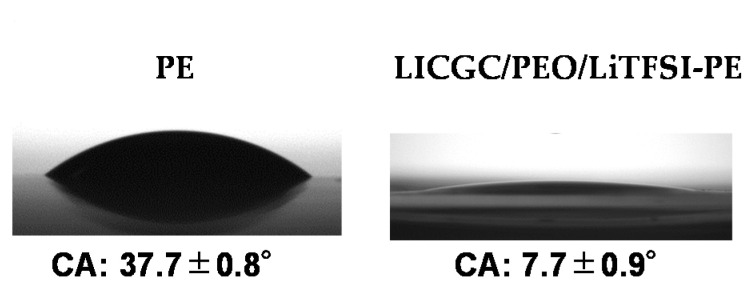
Contact angle measurements of the PE and the LICGC/PEO/LiTFSI-PE separators against the electrolyte.

The organic/inorganic composite solid electrolyte layer was coated on the surface of a commercial PE membrane separator by a simple blade coating method, using an acetonitrile-based slurry containing LICGC particles, PEO, and LiTFSI (LICGC/PEO/LiTFSI = 10/2/1, w/w/w). SEM images of the as-provided PE separator and the LICGC/PEO/LiTFSI-coated PE separator (LICGC/PEO/LiTFSI-PE) are shown in Figure 1a–c. The surface image of coated separator shows uniform distribution of LICGC particles on the separator surface (Figure 1b). In addition, cross-sectional SEM and EDS images confirmed that the formed LICGC/PEO/LiTFSI composite layer was a few microns in thickness (Figure 1c–i). These results indicate that the scalable blade coating approach was able to fabricate the composite electrolyte layer on the PE separator without the aggregation of the ceramic particles. Furthermore, the contact angle measurements for the PE and the LICGC/PEO/LiTFSI-PE separators indicated that the wettability against the electrolyte was significantly improved by introducing the coating layer (Figure 2).

### 3.2. Impedance Measurements of Li/Li Symmetric Cells

**Figure 3:**
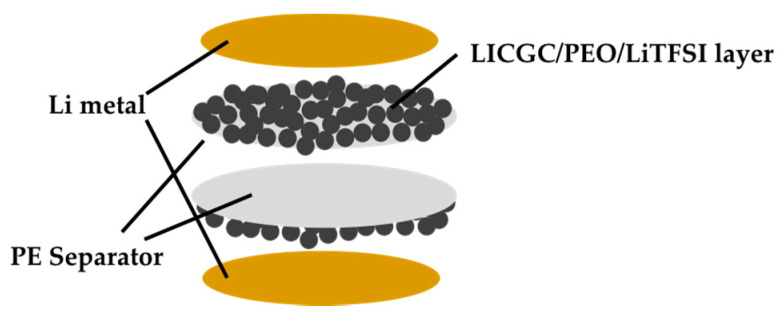
Configuration of the Li/Li symmetric cell using the LICGC/PEO/LiTFSI-PE separator.

**Figure 4:**
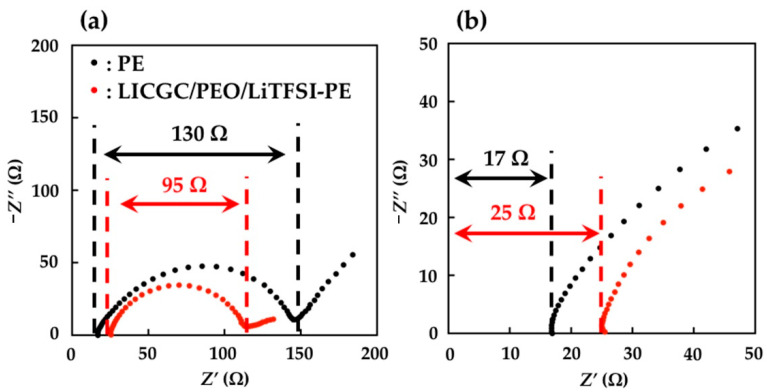
Nyquist plots of the impedance spectra for Li/Li symmetric cells using PE and LICGC/PEO/LiTFSI-PE separators. (**a**) Overview of the impedance spectra and (**b**) expanded spectra in the high-frequency region.

The electrochemical performance of the PE and LICGC/PEO/LiTFSI-PE separators was evaluated using the Li/Li symmetric cell configuration. To ensure a buffer layer between the Li metal electrode and the electrolyte solution, the coated side of the two LICGC/PEO/LiTFSI-PE separators was brought into contact with the Li metal electrode (Figure 3). Figure 4 shows the Nyquist plots of the impedance spectra for the Li/Li cells with PE and LICGC/PEO/LiTFSI-PE separators. Because the coated composite layer was only a few microns thick, the slight difference in thickness between the coated and uncoated PE separators only had a minimal effect on the impedance spectrum. In the impedance spectrum, a high-frequency intercept is related to the bulk resistance of the electrolyte layer, while the depressed semicircular part is considered to be the interfacial resistance between the electrolyte and the Li metal electrode. The cell using the uncoated PE separator showed a bulk resistance of approximately 17 Ω, whereas this value was approximately 25 Ω for the cell using the LICGC/PEO/LiTFSI-PE separator. The slightly higher bulk resistance after coating the separator with LICGC/PEO/LiTFSI could be attributed to the slower diffusion of Li-ion in the composite layer than that in the bulk electrolyte solution. In contrast, the interfacial resistance of the Li/Li cell was significantly suppressed when using the coated separator. This indicates that the inorganic/organic LICGC/PEO/LiTFSI composite electrolyte layer serves as an interfacial buffer to protect the electrolyte solution from directly contacting the Li metal. As a result, the formation of a high-resistance interfacial layer was suppressed, which led to a reduced electrode–electrolyte interfacial resistance.

### 3.3. Li Deposition/Dissolution in Li/Li Symmetric Cells

**Figure 5:**
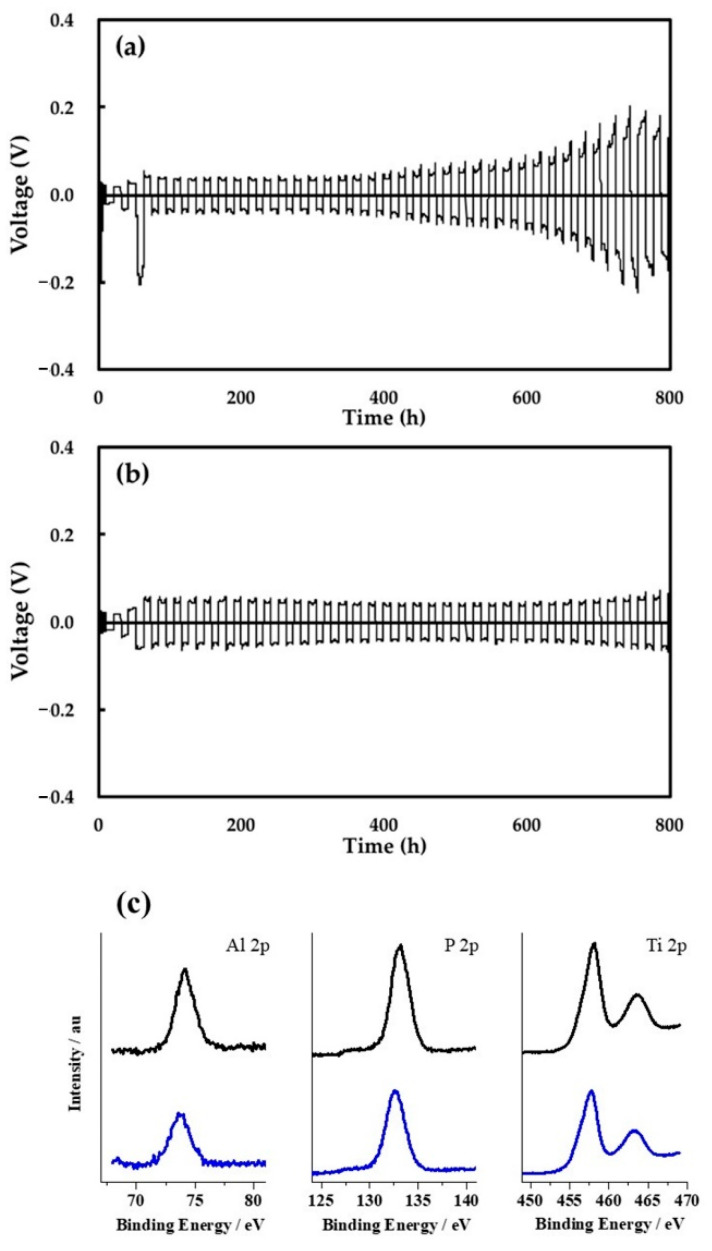
(**a**,**b**) Cycling performance of the Li deposition/dissolution process for the Li/Li symmetric cells using (**a**) the PE separator and (**b**) the LICGC/PEO/LiTFSI-PE separator. (**c**) XPS analysis of the LICGC/PEO/LiTFSI-coated PE separator before (black) and after (blue) 10 cycles of Li deposition/dissolution reaction.

Figure 5 compares the cycling performance of the Li deposition/dissolution process in the Li/Li symmetric cells using the two separators. The voltage drop that occurred at the first cycle at a current density of 0.4 mA/cm^2^ in Figure 5a might be attributed to the irreversible reaction of the native SEI of the lithium metal. For the cell with PE separator, a gradual increase in the cell overpotential was observed after approximately 400 h (20 cycles) at a current density of 0.4 mA/cm^2^ and an areal capacity of 4.0 mAh/cm^2^ (Figure 5a). This change could be ascribed to the undesired reaction of the electrolyte solution with Li metal and the subsequent formation of a highly resistive SEI. On the other hand, such an increase in cell overpotential was not observed when using the LICGC/PEO/LiTFSI-PE separator even after 800 h (40 cycles), indicating higher stability during the Li deposition/dissolution cycles (Figure 5b). We attribute this to the suppression of electrolyte decomposition at the Li metal surface, as well as the formation of a less resistive SEI due to the presence of the organic/inorganic LICGC/PEO/LiTFSI layer. The latter is consistent with the reduced interfacial resistance of the pristine Li/Li cell using the LICGC/PEO/LiTFSI-PE separator (Figure 4a). In addition, the higher mechanical strength of the composite layer could help suppress the formation and growth of dendritic Li during Li deposition/dissolution [38,39,40]. Another possible reason for the improved cycling stability is that a large amount of Li^+^-conducting LICGC particles in the composite layer might regulate Li^+^ diffusion and homogenize Li^+^ flux at the Li metal interface [41]. The excellent long-term cycling performance of the Li/Li symmetric cell confirms the effectiveness of the facile coating strategy of an inorganic/organic solid electrolyte layer for stabilizing Li metal electrodes during battery operation. It also should be noted that LICGC/PEO/LiTFSI-PE separator showed no clear chemical change even after repeated Li deposition/dissolution reaction, which was confirmed by XPS analysis (Figure 5c). In particular, there is a concern that Ti^+4^ in LICGC is to be reduced to Ti^+3^ by contacting with metallic lithium, which largely diminishes the Li-ion conductivity of LICGC. However, the results of XPS analysis clearly revealed that the Ti ion in LICGC remained as Ti^+4^. These results suggest the high stability of the LICGC/PEO/LiTFSI-coated layer.

### 3.4. Morphology of Separator Surface after Li Deposition/Dissolution Test

**Figure 6:**
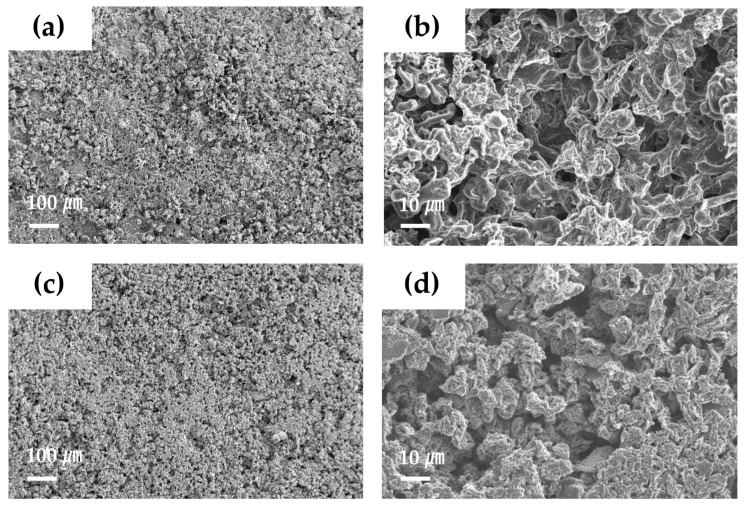
SEM images of the Li metal electrode surface after the Li deposition/dissolution cycling test for the Li/Li symmetric cell (**a**,**b**) with the PE separator and (**c**,**d**) with the LICGC/PEO/LiTFSI-coated PE separator.

SEM observation was used to investigate the surface morphology of the Li metal electrode after the Li deposition/dissolution cycling test (Figure 6). In the cell using the PE separator, the Li surface after cycling showed a rough and uneven structure (Figure 6a,b). This would be related to unstable Li deposition/dissolution during cycling, owing to the formation of a highly resistive SEI layer. On the other hand, the Li surface in the cell using LICGC/PEO/LiTFSI-PE separator showed a relatively uniform morphology (Figure 6c,d). Although this Li surface still contained pores, the overall structure was denser with fewer pores than the case using the PE separator. Therefore, the LICGC/PEO/LiTFSI composite buffer layer between the Li metal electrode and the electrolyte solution likely suppressed the deposition of heterogeneous and highly porous Li during the Li deposition/dissolution reaction. This result is also consistent with the improved cycling stability of the Li/Li symmetric cell using the coated separator (Figure 5b). Thus, introducing the organic/inorganic solid electrolyte layer to the Li metal surface led to uniform Li deposition and consequently stable long-term cycling performance.

### 3.5. Cycling Performance of Li-Oxygen Battery Cells

**Figure 7:**
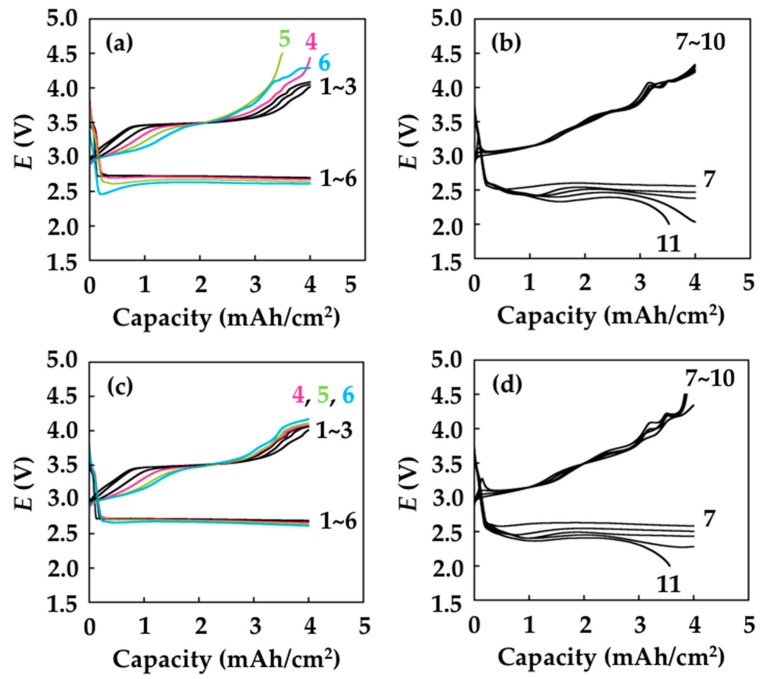
Discharge/charge performance of LOBs. (**a**) Cycles 1–6 and (**b**) cycles 7–11 for the LOB equipped with the PE separator. (**c**) Cycles 1–6 and (**d**) cycles 7–11 for the LOB equipped with the LICGC/PEO/LiTFSI-PE separator.

The results of the repeated Li deposition/dissolution test (Figure 5a,b) and SEM analysis of the electrode (Figure 6) clearly revealed the effectiveness of introducing the LICGC/PEO/LiTFSI coating layer for improving the reversibility of the lithium metal electrode. To demonstrate the effectiveness of the LICGC/PEO/LiTFSI coating layer on the Li metal-based batteries, we performed the discharge/charge cycle test of lithium-oxygen batteries (LOBs), which has the potential to show a higher energy density than conventional lithium-ion batteries. As the mass loading of the coating layer is less than 1 mg/cm^2^, the introduction of the coating layer has limited influence on the energy density of LOBs. Here, the LOB cells have a stacked configuration, and their electrolyte contains redox meditators [42,43]. The discharge/charge performance test was conducted at a current density of 0.4 mA/cm^2^, a capacity limit of 4.0 mAh/cm^2^, and cutoff voltages of 2.0 V/4.5 V. Figure 7a shows the representative voltage profile of the LOB using the uncoated PE separator. During the discharge process, the cell exhibited a voltage plateau at approximately 2.6 V. In the initial and middle parts of the charging process, a stable voltage plateau appeared at approximately 3.5–3.6 V, and the voltage gradually increased to 4.0 V at the end of the charging process. These results are characteristic of the charging profile of LOB cells containing LiNO_3_ and LiBr as redox mediators [44,45]. However, such stable discharge/charge voltages were only maintained up to the 3rd cycle, and the overpotential increased at the end of charging in the 4th~6th cycles. In contrast, the increase in charging voltage was largely suppressed in the LOB equipped with the LICGC/PEO/LiTFSI-coated separator (Figure 7c). Several mechanisms could cause such an increase in the overpotential, such as the accumulation of lithium carbonate-like solid-state side product on the porous carbon positive electrode [46,47] and deterioration of the lithium metal negative electrode [48,49]. Based on the results in Figure 7a,c, we think that the LICGC/PEO/LiTFSI coating on the separator led to more uniform Li deposition, which helped suppress the elevation of overpotential at the end of the charging process. After seven cycles, the LOB cells with and without the LICGC/PEO/LiTFSI coating both showed a gradual decrease in the discharge voltage, which reached the cutoff voltage of 2.0 V in the 11th cycle (Figure 7b,d).

## 4. Conclusions

In this study, a facile and scalable coating method was developed for a commercial polyethylene separator in order to improve the electrochemical performance of lithium metal batteries. An organic/inorganic composite layer containing a lithium-ion-conductive ceramic filler (LICGC) and a solid polymer electrolyte (PEO/LiTFSI) with a thickness of a few microns was coated on the surface of the polyethylene separator via a blade-coating method. We used the commercial PE separator as a substrate separator, but the present coating strategy can be applied to other commercial separators, such as a polypropylene separator. The Li/Li symmetric cell using the LICGC/LEO/LiTFSI-PE separator showed improved interfacial resistance, indicating the formation of a less resistive SEI layer due to the suppression of direct contact between the Li metal and the electrolyte. As a result, long-term stable cycling of Li deposition/dissolution was realized in the cell with the LICGC/PEO/LiTFSI-PE separator. Finally, when the LICGC/PEO/LiTFSI-PE separator was applied in the LOB full cell, the charging overpotential was suppressed. These results indicate that this simple and scalable coating of lithium-ion-conductive fillers composited with solid polymer electrolytes is an effective strategy to improve the electrochemical performance of lithium metal batteries.

## Data Availability

Not applicable.
